# Supplementary material for: Artificial intelligence-enhanced handheld breast ultrasound for screening: A systematic review of diagnostic test accuracy
Source: PLOS Digit Health. 2025 Sep 22;4(9):e0001019. doi: 10.1371/journal.pdig.0001019 (PMC12453205; doi:10.1371/journal.pdig.0001019)
Supplement: S1 File — Complete search strings for PubMed and Google Scholar searches. (PDF) [file pdig.0001019.s001.pdf]

ADDITIONAL FILE 1

The PubMed and Google Scholar searches were conducted with the following (respective) search strings:

("breast"[Title] AND (("US"[Title] OR "ultrasound"[Title] OR "sonogram"[Title] OR "sonograms"[Title] OR "ultrasonograms"[Title] OR "ultrasonogram"[Title] OR "ultrasonography"[Title] OR "sonography"[Title] OR "BUS"[Title]) AND ("ai"[Title] OR "artificial intelligence"[Title] OR "convolution*"[Title] OR "learn*"[Title] OR "transformer"[Title] OR "vit"[Title] OR "deep"[Title] OR "network"[Title] OR "*net"[Title]))) AND 2016/01/01:2023/12/31[Date - Publication]

intitle:"breast" AND ((intitle:"US" OR intitle:"ultrasound" OR intitle:"sonography" OR intitle:"ultrasonography" OR intitle:"sonogram" OR intitle:"ultrasonogram") AND (intitle:"AI" OR intitle:"vit" OR intitle:"transformer" OR intitle:"artificial intelligence" OR intitle:"convolutional" OR intitle:"convolution" OR intitle:"learn" OR intitle:"learned" OR intitle:"learning" OR intitle:"learner" OR intitle:"deep" OR intitle:"vit" OR intitle:"transformer" OR intitle:"network" OR intitle:"net"))

The Google Scholar search also had a date filter applied after searching to limit results to those items dated post 2016.
