# Supplementary material for: Artificial intelligence-enhanced handheld breast ultrasound for screening: A systematic review of diagnostic test accuracy
Source: PLOS Digit Health. 2025 Sep 22;4(9):e0001019. doi: 10.1371/journal.pdig.0001019 (PMC12453205; doi:10.1371/journal.pdig.0001019)
Supplement: S2 File — Complete list and description of data characteristics of public datasets referenced by name in the main text. (PDF) [file pdig.0001019.s002.pdf]

Public BUS dataset summary table. These datasets are referred to by name in the main text.

Dataset	Population	US Machine	Labels	Preprocessing	Availability
BUSI	780 images from 600 patients (26.9% malignancy) from a single hospital in Egypt	LOGIQ E9; LOGIQ E9 Agile	**Classification:** Unclear **Segmentation:** Unclear	Hand-cropped images to remove software artifacts	Public (1)
UDIAT (Dataset B)	163 images from 163 patients (32.5% malignancy) from a single hospital in Spain	Siemens ACUSON Sequoia C512	**Classification:** Unclear **Segmentation:** Delineations from “experienced radiologists”		Public (2)
BUSIS	562 images from ? patients from 3 hospitals in China	GE VIVID 7; LOGIQ E9; Hitachi EUB-6500; Philips iU2; Siemens ACUSON S2000	**Segmentation:** Delineations from agreement between 3 radiologists with final judgement by 4th radiologist		Public (3)
OASBUD	200 images from 78 patients (52% malignancy) from a single hospital in Poland	Ultrasonix SonixTouch	**Segmentation:** Delineations (ROI) from 1 radiologist **Classification:** Histological results from biopsy with benign follow-up of at least 2 years		Public (4)
ultrasoundcases	857 images from ? patients from a single hospital in the Netherlands	Fujifilm Ultrasound System	**Classification:** Histological results from biopsy		Public (5)
STU-Hospital	42 images from ? patients from a single hospital in China	GE Voluson E10	**Segmentation:** Unclear		Public (6)
Mendeley	250 images from 50 patients (60% malignancy)		**Classification:** Histological results from biopsy		Public (7)
onlinemedicalimages	181 images from ? patients (33.1% malignancy) from a single hospital in Thailand	Philips iU22	**Classification:** Unclear		Public (8)
radiopaedia	504 images from ? patients (51.9% malignancy) from ?		**Classification:** Histological results from biopsy		Public (9)

i

1. Al-Dhabyani W, Gomaa M, Khaled H, Fa

hmy A. Dataset of breast ultrasound images. Data in brief. 2020;28:104863.

2. Yap MH, Pons G, Marti J, Ganau S, Sentis M, Zwiggelaar R, Davison AK, Marti R. Automated Breast Ultrasound Lesions Detection Using Convolutional Neural Networks. IEEE Journal of Biomedical and Health Informatics. 2018;22(4):1218-26. doi: 10.1109/jbhi.2017.2731873.

3. Zhang Y, Xian M, Cheng H-D, Shareef B, Ding J, Xu F, Huang K, Zhang B, Ning C, Wang Y. BUSIS: A Benchmark for Breast Ultrasound Image Segmentation. Healthcare. 2022;10(4):729. doi: 10.3390/healthcare10040729.

4. Piotrzkowska-Wróblewska H, Dobruch-Sobczak K, Byra M, Nowicki A. Open access database of raw ultrasonic signals acquired from malignant and benign breast lesions. Med Phys. 2017;44(11):6105-9. Epub 20170925. doi: 10.1002/mp.12538. PubMed PMID: 28859252.

5. Geertsma T. Ultrasound Cases. Available from: https://www.ultrasoundcases.info/cases/breast-and-axilla/.

6. Zhuang Z, Li N, Joseph Raj AN, Mahesh VG, Qiu S. An RDAU-NET model for lesion segmentation in breast ultrasound images. PloS one. 2019;14(8):e0221535.

7. Rodrigues PS. Breast Ultrasound Image. Mendeley.

8. Ultrasound Images. Available from: http://onlinemedicalimages.com/.

9. Cases. Available from: https://radiopaedia.org/.

---

i
