# Supplementary material for: Artificial intelligence-enhanced handheld breast ultrasound for screening: A systematic review of diagnostic test accuracy
Source: PLOS Digit Health. 2025 Sep 22;4(9):e0001019. doi: 10.1371/journal.pdig.0001019 (PMC12453205; doi:10.1371/journal.pdig.0001019)
Supplement: S3 File — QUality Assessment of Diagnostic Accuracy Studies-2 (QUADAS-2) criteria used to assess the risk of bias for this systematic review, adapted from [14]. (PDF) [file pdig.0001019.s003.pdf]

QUADAS-2 Criteria

Item	Response
**PARTICIPANT SELECTION – A. RISK OF BIAS**	
Was a consecutive or random sample of patients enrolled?	**Yes –** RCTs and cohort studies (prospective or retrospective) with unenriched (consecutive or random) sampling<br>**Unclear –** If not stated<br>**No –** Other studies
Did the study avoid inappropriate exclusions?	**Yes –** If inappropriate exclusions were avoided<br>**Unclear –** If not clearly reported<br>**No –** Exclusion of more than 10% of the samples for any reason, for example retrospective studies with missing data<br>**No –** Systematic exclusion of types of women/images (i.e., of dense breasts, breast implants)<br>**No –** Exclusion based on outcomes (e.g., exclusion of cancer types, exclusion of interval cancers, exclusion/inclusion based on recall decision)
Were the women and breast US exams included in the results reported independent of those used to train the AI algorithm?	**Yes –** External geographical validation (test set was sampled from a different center; can be in another country or the same country). Studies which sample from a geographically external center within the same immediate geographical area are noted as having an elevated risk of bias.<br>**Unclear –** No details stated about the geographical sources of the training set and tuning set.<br>**No –** Any internal validation (e.g., split sample) or temporal validation
**PARTICIPANT SELECTION – B. CONCERNS REGARDING APPLICABILITY**	
Is there concern that the included patients do not match the review question?	**High –** If "yes" for any of the following statements<br>**Unclear –** If no details are provided<br>**Low –** If "no" for all the following statements<br>• Not a consecutive or random sample<br>• Enriched sample/cancer prevalence doesn't match the screening context (>3%)<br>• Women/women's BUS exams are from a racially homogeneous (>75% from same racial group) or unspecified population<br>• Women/women's BUS exams are from the same BUS system (>75% from the same BUS examination system)
**INDEX TESTS – A. RISK OF BIAS**	
Were the index tests interpreted without knowledge of the results of the reference standard?	For index tests where a human is involved (either human read comparator or AI as reader aid):<br><br>**Yes -** Require clear statement of blinding, or clear temporal relationships where the human read occurred before the reference standard (for

	segmentation algorithms, readers were blinded to reference standard delineations)<br>**No -** Otherwise<br><br>For index tests where AI is used without any human element:<br><br>**Yes -** AI system has not previously been trained on these BUS images or learned from these BUS images or other BUS images from the same women<br>**Unclear -** If not explicit that there has been no repeat within same or previous studies<br>**No -** If any repeat use of the same cases
Were the index test results interpreted without knowledge of the results of any other index tests?	For index tests where a human is involved (either human read comparator or AI as reader aid):<br><br>**No -** If human readers were not blinded to AI (unless that AI is specifically part of the same index test)<br>**No -** If AI systems are trained or calibrated using decisions from human readers in same cases (for segmentation algorithms, this means different readers or a wash-out period between labeling/scoring reads)<br>**Yes –** Otherwise<br><br>For index tests where AI is used without any human element:<br><br>**No –** If AI results were interpreted in combination with labels or another AI algorithm (i.e., choosing the “lowest common denominator” between the label and the prediction)<br>**Yes –** Otherwise
If a threshold as used, was it pre-specified?	**Yes -** For systems giving a risk score where the study explicitly states the pre-specified threshold<br>**No -** Using sensitivity / specificity of the reader as benchmark using the same dataset<br>**No -** Setting the threshold with the validation set without temporal evidence (e.g., published protocol) that threshold was truly pre-specified<br>**NA -** Human readers or human/AI combinations, or no threshold specified<br>**NA –** Segmentation-only studies without a reader study
Where human readers are part of the test, were their decisions made in a clinical practice context? (i.e., avoidance of the laboratory effect)	**Yes –** If the readers made decisions in the clinical context, and those decisions were used to decide whether to biopsy or recall women (either prospectively as part of a trials or test accuracy study or retrospective studies using the original decision)<br>**No –** If the readers examined a test set (of any prevalence) outside of clinical practice, or any other context likely to result in the laboratory effect

	**NA –** If no human readers involved
**INDEX TESTS – B. CONCERNS REGARDING APPLICABILITY**	
Is there concern that the index test(s) or comparator, its conduct, or interpretation differ from the review question?	**High –** If "yes" for any of the following<br>**Unclear –** If no details are provided<br>**Low –** If "no" for all the following<br>• Not a complete testing pathway applicable to clinical practice (for example AI accuracy for a single image, but not aggregated into patient-, breast-, or mass-level decisions)<br>• AI system/reader had no access to prior exams, all images from exam, or incomplete exams available
**REFERENCE STANDARD – A. RISK OF BIAS**	
Is the reference standard likely to correctly classify the target condition?	<u>For classification studies</u><br>**Yes –** If the reference standard is histopathology results from biopsy with at least 2 years follow-up to interval cancers<br>**No –** If the reference standard is histopathology results from biopsy with no follow-up. Retrospective studies which fail to report follow-up protocol are considered as missing follow-up.<br><br><u>For segmentation studies</u><br>**Yes –**If the reference standard is agreement in delineation results from >1 physician with experience in breast ultrasound.<br>**Unclear –** If the reference standard is delineations automatically or semi-automatically generated by other software.<br>**No –** Otherwise<br><br><u>For segmentation and classification studies</u><br>**Yes –** "Yes" conditions are met for both segmentation and classification reference standard as defined above.<br>**No –** Otherwise.<br><br><u>For detection studies</u><br>**Yes –** (for models which provide segmentation mask-style detections) "Yes" conditions are met for both segmentation and classification reference standard as defined above.<br>**Yes –** (for models which provide bounding box-style detections) "Yes" condition is met for classification reference standard as defined above and lesion location is defined as agreement in lesion location from >1 physician with experience in breast ultrasound.<br>**No –** Otherwise

	For frame selection studies<br>**Yes –**If the reference standard is agreement in responsible frame selection from >1 physician with experience in breast ultrasound.<br>**No –** Otherwise
Were the reference standard results interpreted without knowledge of the results of the index test?	**Yes –** Retrospective studies where labels are created wholly before AI development<br>**Yes –** Retrospective studies where readers read exams prospectively<br>**No –** For prospective studies if the investigators did not blind the clinicians undertaking the follow-up tests to which index test examined the exams, for example by putting location marks in the same format for AI and human readers
**REFERENCE STANDARD – B. CONCERNS REGARDING APPLICABILITY**	
Is there concern that the target condition as defined by the reference standard does not match the review question?	**High –** If "yes" for any of the following<br>**Unclear –** If no details are provided<br>**Low –** If "no" for all of the following<br>• Length of screening rounds <2 years for follow-up/definition of interval cancers<br>• Classification not by biopsy/follow-up (i.e., by physician-assigned BI-RADS scores)<br>• Segmentation assigned by assessment from <2 physicians<br>• Lesion location assigned by assessment from <2 physicians<br>• Responsible frame decision assigned by assessment from <2 physicians
**FLOW & TIMING – A. RISK OF BIAS**	
Did all patients receive a reference standard?	**No –** If there was significant (>10%) loss to follow-up for reference standards of interval cancers or subsequent screening results<br>**No –** If any women who should have received a biopsy after index test positive results did not receive one or results were unavailable.<br>**NA –** Studies where the reference standard is not related to presence/absence of breast cancer diagnosis (i.e., segmentation and frame selection)<br>**Yes –** Otherwise. Classification-only AI methods which use only biopsy (no follow-up) as their reference standard are included in this group.
Did the study avoid choosing which reference standard based on results of just one of the index tests?	**Yes –** For test-treat RCTs randomizing to different test strategies and their associated recall decisions<br>**Yes –** If women testing positive in any of the included index tests (AI pathways or comparator human pathways) all receive follow-up tests/biopsy in a prospective study. AI methods which use only biopsy (no follow-up) as their reference standard are included in this group.

	**No –** If women were recalled for further tests on the basis of one of the index tests (either AI or human) and not the others then this will cause bias<br>**No –** In retrospective studies, the decision whether to recall for follow-up tests/biopsy was made on the basis of the human readers' decision.<br>**Unclear –** Segmentation and frame selection studies.
Were all patients included in the analysis?	**Yes –** Otherwise.<br>**No –** If there were any exclusions after the point of selecting the cohort, for example intermediate or indeterminate results
