# Supplementary material for: Artificial intelligence-enhanced handheld breast ultrasound for screening: A systematic review of diagnostic test accuracy
Source: PLOS Digit Health. 2025 Sep 22;4(9):e0001019. doi: 10.1371/journal.pdig.0001019 (PMC12453205; doi:10.1371/journal.pdig.0001019)
Supplement: S6 File — Complete list of studies excluded after full-text review with reasons for exclusion (after adjudication). (PDF) [file pdig.0001019.s006.pdf]

Full-Text Exclusions and Reason for Exclusion (Post-Adjudication)

Title	Year	Final Decision
Deep learning-based multimodal fusion network for segmentation and classification of breast cancers using B-mode and elastography ultrasound images.	2023	MULTIMODAL
Development of a Deep Learning-Based Model for Diagnosing Breast Nodules With Ultrasound.	2021	UNCLEAR SPLIT
Diagnostic Performance of an Artificial Intelligence System in Breast Ultrasound.	2022	VALIDATION
Automatic tumor segmentation in breast ultrasound images using a dilated fully convolutional network combined with an active contour model.	2019	UNCLEAR SPLIT
Breast ultrasound lesion classification based on image decomposition and transfer learning.	2020	NOT PATIENT SPLIT
Breast ultrasound image segmentation: A coarse-to-fine fusion convolutional neural network.	2021	NOT PATIENT SPLIT
FMRNet: A fused network of multiple tumoral regions for breast tumor classification with ultrasound images.	2022	NOT PATIENT SPLIT
Mssa-net: Multi-scale self-attention network for breast ultrasound image segmentation	2021	NOT PATIENT SPLIT
Automatic identification of breast ultrasound image based on supervised block-based region segmentation algorithm and features combination migration deep …	2019	2 SPLIT
Breast UltraSound Image classification using fuzzy-rank-based ensemble network	2023	NOT PATIENT SPLIT
BI-RADS-NET-V2: A Composite Multi-Task Neural Network for Computer-Aided Diagnosis of Breast Cancer in Ultrasound Images With Semantic and Quantitative …	2023	NOT PATIENT SPLIT
A temporal sequence dual-branch network for classifying hybrid ultrasound data of breast cancer	2020	UNCLEAR SPLIT
BCDNet: An Optimized Deep Network for Ultrasound Breast Cancer Detection	2023	NOT PATIENT SPLIT
SEGMENTATION OF CANCER MASSES ON BREAST ULTRASOUND IMAGES USING MODIFIED U-NET	2023	2 SPLIT
Breast tumor ultrasound image classification method based on the fusion of deep network features and texture morphological features	2021	UNCLEAR SPLIT
Segmentation of Breast Cancer on Ultrasound Images using Attention U-Net Model	2023	2 SPLIT
Efficient-Net ASPP Deep Network for Malignant Ultrasound Breast Cancer Segmentation	2023	NOT PATIENT SPLIT
Multilevel Perception Boundary-guided Network for Breast Lesion Segmentation in Ultrasound Images	2023	NOT PATIENT SPLIT
Breast ultrasound image classification and segmentation using convolutional neural networks	2018	UNCLEAR SPLIT
Using convolutional neural networks with direct acyclic graph architecture in segmentation of breast lesions in US images	2019	2 SPLIT
Lesion detection in breast ultrasound images using a machine learning approach and genetic optimization	2019	UNCLEAR SPLIT
… the Performance of Convolutional Neural Networks with Direct and Sequential Acyclic Graph Architectures in Automatic Segmentation of Breast Lesions in Ultrasound …	2020	UNCLEAR SPLIT
Masked Video Modeling with Correlation-Aware Contrastive Learning for Breast Cancer Diagnosis in Ultrasound	2022	UNCLEAR SPLIT
Breast tumor detection in ultrasound images using deep learning	2017	NOT PATIENT SPLIT
Breast Ultrasound Tumor Detection Based on Active Learning and Deep Learning	2022	NOT PATIENT SPLIT
A Novel Transfer Learning-Based Model for Ultrasound Breast Cancer Image Classification	2023	2 SPLIT

Automatic classification of ultrasound breast lesions using a deep convolutional neural network mimicking human decision-making.	2019	METRIC
One step further into the blackbox: a pilot study of how to build more confidence around an AI-based decision system of breast nodule assessment in 2D ultrasound.	2021	BIRADS
Clinical value of radiomics and machine learning in breast ultrasound: a multicenter study for differential diagnosis of benign and malignant lesions.	2021	2 SPLIT
Graph neural network-based breast cancer diagnosis using ultrasound images with optimized graph construction integrating the medically significant features.	2023	NOT PATIENT SPLIT
A gated convolutional neural network for classification of breast lesions in ultrasound images	2022	2 SPLIT
Hybrid deep learning and genetic algorithms approach (HMB-DLGAHA) for the early ultrasound diagnoses of breast cancer	2022	NOT PATIENT SPLIT
Diagnostic Efficiency of the Breast Ultrasound Computer-Aided Prediction Model Based on Convolutional Neural Network in Breast Cancer.	2020	UNCLEAR SPLIT
Application of Artificial Intelligence Computer-Assisted Diagnosis Originally Developed for Thyroid Nodules to Breast Lesions on Ultrasound.	2022	NOT PATIENT SPLIT
Breast Tumor Classification in Ultrasound Images by Fusion of Deep Convolutional Neural Network and Shallow LBP Feature.	2023	2 SPLIT
BUS-Net: Breast Tumour Detection Network for Ultrasound Images Using Bi-directional ConvLSTM and Dense Residual Connections.	2023	NOT PATIENT SPLIT
Improved U-net MALF model for lesion segmentation in breast ultrasound images	2021	NOT PATIENT SPLIT
Neural Network Pattern Recognition of Ultrasound Image Gray Scale Intensity Histograms of Breast Lesions to Differentiate Between Benign and Malignant …	2021	NOT PATIENT SPLIT
Breast tumor segmentation in ultrasound images based on u-NET model	2022	2 SPLIT
Breast lesion segmentation and characterization using the Small Tumor-Aware Network (STAN) and 2D/3D shape descriptors in ultrasound images	2021	2 SPLIT
EfficientU-net: a novel deep learning method for breast tumor segmentation and classification in ultrasound images	2023	NOT PATIENT SPLIT
DG-CNN: Introducing margin information into convolutional neural networks for breast cancer diagnosis in ultrasound images	2022	2 SPLIT
Fully multi-target segmentation for breast ultrasound image based on fully convolutional network.	2020	2 SPLIT
Transfer learning for automatic joint segmentation of thyroid and breast lesions from ultrasound images.	2022	UNCLEAR SPLIT
Evaluating different combination methods to analyse ultrasound and shear wave elastography images automatically through discriminative convolutional neural network in breast cancer imaging.	2022	NOT PATIENT SPLIT
A hybrid attentional guidance network for tumors segmentation of breast ultrasound images.	2023	2 SPLIT
Distinction between benign and malignant breast masses at breast ultrasound using deep learning method with convolutional neural network.	2019	2 SPLIT
Improving the classification performance of breast ultrasound image using deep learning and optimization algorithm	2021	NOT PATIENT SPLIT
A Multi-attention Triple Decoder Deep Convolution Network for Breast Cancer Segmentation Using Ultrasound Images	2023	2 SPLIT
Achieving highly efficient breast ultrasound tumor classification with deep convolutional neural networks	2022	NOT PATIENT SPLIT
U-Net and SegNet performances on lesion segmentation of breast ultrasonography images	2021	NOT PATIENT SPLIT
Semi-supervised segmentation of lesion from breast ultrasound images with attentional generative adversarial network.	2020	
Computer-aided diagnosis of breast ultrasound images using ensemble learning from convolutional neural networks.	2020	NOT PATIENT SPLIT
C-Net: Cascaded convolutional neural network with global guidance and refinement residuals for breast ultrasound images segmentation.	2022	NOT PATIENT SPLIT
A comparative study of pre-trained convolutional neural networks for semantic segmentation of breast tumors in ultrasound.	2020	NOT PATIENT SPLIT

ATFE-Net: Axial Transformer and Feature Enhancement-based CNN for ultrasound breast mass segmentation.	2023	NOT PATIENT SPLIT
HCTNet: A hybrid CNN-transformer network for breast ultrasound image segmentation.	2023	NOT PATIENT SPLIT
Classification of malignant tumors in breast ultrasound using a pretrained deep residual network model and support vector machine.	2021	NOT PATIENT SPLIT
Deep learning-based classification of breast lesions using dynamic ultrasound video.	2023	NOT PATIENT SPLIT
The effect of image resolution on convolutional neural networks in breast ultrasound.	2023	TASK
Breast cancer classification based on convolutional neural network and image fusion approaches using ultrasound images.	2023	NOT PATIENT SPLIT
Dual-mode artificially-intelligent diagnosis of breast tumours in shear-wave elastography and B-mode ultrasound using deep polynomial networks.	2019	UNCLEAR SPLIT
Automated diagnosis of breast ultrasonography images using deep neural networks.	2019	NOT PATIENT SPLIT
Global guidance network for breast lesion segmentation in ultrasound images.	2021	NOT PATIENT SPLIT
Deep weakly-supervised breast tumor segmentation in ultrasound images with explicit anatomical constraints.	2022	NOT PATIENT SPLIT
A generic deep learning framework to classify thyroid and breast lesions in ultrasound images.	2021	THYROID
Joint segmentation and classification of breast masses based on ultrasound radio-frequency data and convolutional neural networks.	2022	NOT PATIENT SPLIT
Ultrasound classification of breast masses using a comprehensive Nakagami imaging and machine learning framework.	2022	NOT PATIENT SPLIT
Computer-Aided Diagnosis for Breast Ultrasound Using Computerized BI-RADS Features and Machine Learning Methods.	2016	2 SPLIT
Attention-Enriched Deep Learning Model for Breast Tumor Segmentation in Ultrasound Images.	2020	NOT PATIENT SPLIT
Deep Learning for Differentiation of Breast Masses Detected by Screening Ultrasound Elastography.	2023	NOT PATIENT SPLIT
Deep learning-based computer-aided diagnosis in screening breast ultrasound to reduce false-positive diagnoses.	2021	FEATURES
Classification of malignant tumours in breast ultrasound using unsupervised machine learning approaches.	2021	UNCLEAR SPLIT
Breast lesion detection using an anchor-free network from ultrasound images with segmentation-based enhancement.	2022	NOT PATIENT SPLIT
Computer-Aided Diagnosis with Deep Learning Architecture: Applications to Breast Lesions in US Images and Pulmonary Nodules in CT Scans.	2016	CT
Computer-aided diagnosis system for breast ultrasound images using deep learning.	2019	NOT PATIENT SPLIT
A novel approach with dual-sampling convolutional neural network for ultrasound image classification of breast tumors.	2020	2 SPLIT
Automatic Detection of Breast Calcification in Ultrasound Imaging with Convolutional Neural Network	2021	NOT PATIENT SPLIT
Comparison of two Deep Learning Methods for Classification of Dataset of Breast Ultrasound Images	2021	2 SPLIT
Improving breast cancer diagnosis by incorporating raw ultrasound parameters into machine learning.	2022	2 SPLIT
Deep learning applied to two-dimensional color Doppler flow imaging ultrasound images significantly improves diagnostic performance in the classification of breast masses: a multicenter study.	2021	METRIC
Deep learning applied to two-dimensional color Doppler flow imaging ultrasound images significantly improves diagnostic performance in the classification of breast …	2021	DUPLICATE
Added value of deep learning-based computer-aided diagnosis and shear wave elastography to b-mode ultrasound for evaluation of breast masses detected by screening ultrasound.	2021	BIRADS

BI-RADS-NET-V2: A Composite Multi-Task Neural Network for Computer-Aided Diagnosis of Breast Cancer in Ultrasound Images With Semantic and Quantitative Explanations.	2023	NOT PATIENT SPLIT
Ultrasound-Based Diagnosis of Breast Tumor with Parameter Transfer Multilayer Kernel Extreme Learning Machine.	2019	NO SPLIT
Deep Learning Networks for Breast Lesion Classification in Ultrasound Images: A Comparative Study.	2023	ABSTRACT ONLY
MSF-GAN: Multi-Scale Fuzzy Generative Adversarial Network for Breast Ultrasound Image Segmentation.	2021	UNCLEAR SPLIT
Comparative Analysis of Current Deep Learning Networks for Breast Lesion Segmentation in Ultrasound Images.	2022	NOT PATIENT SPLIT
STAN: SMALL TUMOR-AWARE NETWORK FOR BREAST ULTRASOUND IMAGE SEGMENTATION.	2020	2 SPLIT
Automated Breast Ultrasound Lesions Detection Using Convolutional Neural Networks.	2018	2 SPLIT
Joint Localization and Classification of Breast Cancer in B-Mode Ultrasound Imaging via Collaborative Learning With Elastography.	2022	2 SPLIT
Boundary-Guided and Region-Aware Network With Global Scale-Adaptive for Accurate Segmentation of Breast Tumors in Ultrasound Images.	2023	2 SPLIT
BI-RADS-NET: AN EXPLAINABLE MULTITASK LEARNING APPROACH FOR CANCER DIAGNOSIS IN BREAST ULTRASOUND IMAGES.	2021	NOT PATIENT SPLIT
MDF-Net: A Multi-Scale Dynamic Fusion Network for Breast Tumor Segmentation of Ultrasound Images.	2023	2 SPLIT
Joint Weakly and Semi-Supervised Deep Learning for Localization and Classification of Masses in Breast Ultrasound Images.	2019	2 SPLIT
Domain Knowledge Powered Deep Learning for Breast Cancer Diagnosis Based on Contrast-Enhanced Ultrasound Videos.	2021	METRIC
HoVer-Trans: Anatomy-Aware HoVer-Transformer for ROI-Free Breast Cancer Diagnosis in Ultrasound Images.	2023	UNCLEAR SPLIT
Feature Pyramid Nonlocal Network With Transform Modal Ensemble Learning for Breast Tumor Segmentation in Ultrasound Images.	2021	NOT PATIENT SPLIT
Machine learning to improve breast cancer diagnosis by multimodal ultrasound.	2018	UNCLEAR SPLIT
Breast ultrasound tumour classification: A Machine Learning—Radiomics based approach	2021	NOT PATIENT SPLIT
Optimal deep transfer learning driven computer-aided breast cancer classification using ultrasound images		UNCLEAR SPLIT
Validating racial and ethnic non-bias of artificial intelligence decision support for diagnostic breast ultrasound evaluation.	2023	METRIC
Classification of breast masses in ultrasound images using self-adaptive differential evolution extreme learning machine and rough set feature selection.	2017	2 SPLIT
Breast ultrasound lesions recognition: end-to-end deep learning approaches.	2019	NOT PATIENT SPLIT
Characterization of indeterminate breast lesions on B-mode ultrasound using automated machine learning models.	2020	UNCLEAR SPLIT
End-to-end breast ultrasound lesions recognition with a deep learning approach	2018	DUPLICATE
Weakly-supervised US breast tumor characterization and localization with a box convolution network	2020	NOT PATIENT SPLIT
Ultrasound image augmentation by tumor margin appending for robust deep learning based breast lesion classification	2022	NOT PATIENT SPLIT
Breast Ultrasound Image Analysis based on Transfer Learning	2020	2 SPLIT
Sparse Representation Based Multi-Instance Learning for Breast Ultrasound Image Classification.	2017	NOT BUS
Is Intensity Inhomogeneity Correction Useful for Classification of Breast Cancer in Sonograms Using Deep Neural Network?	2018	NOT PATIENT SPLIT
Convolutional Neural Network for Breast and Thyroid Nodules Diagnosis in Ultrasound Imaging.	2020	THYROID

CT-ML: Diagnosis of Breast Cancer Based on Ultrasound Images and Time-Dependent Feature Extraction Methods Using Contourlet Transformation and Machine Learning.	2022	RETRACTED
Breast Tumor Ultrasound Image Segmentation Method Based on Improved Residual U-Net Network.	2022	NOT BUS
Artificial Intelligence Medical Ultrasound Equipment: Application of Breast Lesions Detection.	2020	2 SPLIT
Classification of Breast Masses on Ultrasound Shear Wave Elastography using Convolutional Neural Networks.	2020	2 SPLIT
An experimental study on breast lesion detection and classification from ultrasound images using deep learning architectures.	2019	BIRADS
Evaluating the performance of convolutional neural networks with direct acyclic graph architectures in automatic segmentation of breast lesion in US images.	2019	UNCLEAR SPLIT
Artificial intelligence for non-mass breast lesions detection and classification on ultrasound images: a comparative study.	2023	SUBTYPE
Fus2Net: a novel Convolutional Neural Network for classification of benign and malignant breast tumor in ultrasound images.	2021	NOT PATIENT SPLIT
LRSCnet: Local Reference Semantic Code learning for breast tumor classification in ultrasound images.	2021	UNCLEAR SPLIT
Performance of machine learning software to classify breast lesions using BI-RADS radiomic features on ultrasound images.	2019	UNCLEAR SPLIT
No sonographer, no radiologist: Assessing accuracy of artificial intelligence on breast ultrasound volume sweep imaging scans.	2022	SUBTYPE
Automatic semantic segmentation of breast tumors in ultrasound images based on combining fuzzy logic and deep learning-A feasibility study.	2021	NOT PATIENT SPLIT
Segmentation and recognition of breast ultrasound images based on an expanded U-Net.	2021	UNCLEAR SPLIT
CTG-Net: Cross-task guided network for breast ultrasound diagnosis.	2022	UNCLEAR SPLIT
DBU-Net: Dual branch U-Net for tumor segmentation in breast ultrasound images.	2023	NOT PATIENT SPLIT
Explaining a Deep Learning Based Breast Ultrasound Image Classifier with Saliency Maps.	2022	BIRADS
A deep learning-based diagnostic pattern for ultrasound breast imaging: can it reduce unnecessary biopsy?	2022	2 SPLIT
Detection and recognition of ultrasound breast nodules based on semi-supervised deep learning: a powerful alternative strategy.	2021	NOT PATIENT SPLIT
Evaluation of the performance of traditional machine learning algorithms, convolutional neural network and AutoML Vision in ultrasound breast lesions classification: a comparative study.	2021	NOT PATIENT SPLIT
Dilated transformer: residual axial attention for breast ultrasound image segmentation.	2022	NOT PATIENT SPLIT
Machine Learning Models to Improve the Differentiation Between Benign and Malignant Breast Lesions on Ultrasound: A Multicenter External Validation Study.	2021	ABUS
Artificial Intelligence-Based Breast Cancer Diagnosis Using Ultrasound Images and Grid-Based Deep Feature Generator.	2022	NOT PATIENT SPLIT
BGM-Net: Boundary-Guided Multiscale Network for Breast Lesion Segmentation in Ultrasound.	2021	2 SPLIT
Deep Learning-Based Radiomics of B-Mode Ultrasonography and Shear-Wave Elastography: Improved Performance in Breast Mass Classification.	2020	SUBTYPE
Enhancing Performance of Breast Ultrasound in Opportunistic Screening Women by a Deep Learning-Based System: A Multicenter Prospective Study.	2022	BIRADS
BUSnet: A Deep Learning Model of Breast Tumor Lesion Detection for Ultrasound Images.	2022	2 SPLIT
Contrastive learning-guided multi-meta attention network for breast ultrasound video diagnosis.	2022	NOT PATIENT SPLIT
Deep learning algorithm using bispectrum analysis energy feature maps based on ultrasound radiofrequency signals to detect breast cancer	2023	UNCLEAR SPLIT

Artificial intelligence breast ultrasound and handheld ultrasound in the BI-RADS categorization of breast lesions: A pilot head to head comparison study in screening program.	2022	ABUS
Rapid Segmentation and Diagnosis of Breast Tumor Ultrasound Images at the Sonographer Level Using Deep Learning.	2023	2 SPLIT
Ensemble Deep-Learning-Enabled Clinical Decision Support System for Breast Cancer Diagnosis and Classification on Ultrasound Images.	2022	UNCLEAR SPLIT
Dual-Intended Deep Learning Model for Breast Cancer Diagnosis in Ultrasound Imaging.	2022	NOT PATIENT SPLIT
Tumor Segmentation in Breast Ultrasound Image by Means of Res Path Combined with Dense Connection Neural Network.	2021	2 SPLIT
Presentation of Novel Architecture for Diagnosis and Identifying Breast Cancer Location Based on Ultrasound Images Using Machine Learning.	2021	NOT PATIENT SPLIT
Incorporating the Breast Imaging Reporting and Data System Lexicon with a Fully Convolutional Network for Malignancy Detection on Breast Ultrasound.	2021	2 SPLIT
A Machine Learning Ensemble Based on Radiomics to Predict BI-RADS Category and Reduce the Biopsy Rate of Ultrasound-Detected Suspicious Breast Masses.	2022	NOT PATIENT SPLIT
Predicting Breast Tumor Malignancy Using Deep ConvNeXt Radiomics and Quality-Based Score Pooling in Ultrasound Sequences.	2022	2 SPLIT
Edge-Driven Multi-Agent Reinforcement Learning: A Novel Approach to Ultrasound Breast Tumor Segmentation.	2023	UNCLEAR SPLIT
ESTAN: Enhanced Small Tumor-Aware Network for Breast Ultrasound Image Segmentation.	2022	2 SPLIT
A Novel Fuzzy Relative-Position-Coding Transformer for Breast Cancer Diagnosis Using Ultrasonography.	2023	2 SPLIT
An Edge-Based Selection Method for Improving Regions-of-Interest Localizations Obtained Using Multiple Deep Learning Object-Detection Models in Breast Ultrasound Images.	2022	METRIC
A Study on the Effectiveness of Deep Learning-Based Anomaly Detection Methods for Breast Ultrasonography.	2023	TASK
Breast Ultrasound Images Augmentation and Segmentation Using GAN with Identity Block and Modified U-Net 3.	2023	NOT PATIENT SPLIT
Enhancing Breast Cancer Detection through Ultrasound Images using Convolutional Neural Networks (CNNs)	2023	UNCLEAR SPLIT
[Application of multiple empirical kernel mapping ensemble classifier based on self-paced learning in ultrasound-based computer-aided diagnosis for breast cancer].	2021	METRIC
Transfer learning for breast cancer classification using small dataset of ultrasound images	2020	NOT PATIENT SPLIT
Recognition of Benign and Malignant Breast Ultrasound Images Based on Deep Transfer Learning	2021	2 SPLIT
BUS-net: a bimodal ultrasound network for breast cancer diagnosis	2022	NOT PATIENT SPLIT
Breast cancer classification in ultrasound images using transfer learning	2019	NOT PATIENT SPLIT
Computer-Aided Diagnosis System for Breast Ultrasound Reports Generation and Classification Method Based on Deep Learning	2023	TASK
Machine Learning Models for Breast Lesions Based on Ultrasound Imaging Features: A Observational Study	2020	ABUS
Standardized Transfer Learning Models Enhance Classification of Breast Ultrasound Data	2023	2 SPLIT
Bi-modal ultrasound breast cancer diagnosis via multi-view deep neural network svm	2020	2 SPLIT
Transformer-based Characterization of Breast Lesions in Handheld Ultrasound Images WITH Classification Inconsistency Measure	2023	SUBTYPE
Research Article Application of Artificial Neural Network Models in Segmentation and Classification of Nodules in Breast Ultrasound Digital Images	2016	UNCLEAR SPLIT
Application value of deep learning ultrasound in the four-category classification of breast masses	2020	UNAVAILABLE

Video-Based Ultrasound Diagnostic Performance for Breast Cancer Using Deep Learning: A Retrospective, Multicenter, Diagnostic Study		BIRADS
Computer-aided diagnosis of breast ultrasound images using ensemble learning from convolutional neural networks	2020	2 SPLIT
… of the performance of traditional machine learning algorithms, convolutional neural network and AutoML Vision in ultrasound breast lesions classification: A …	2021	NOT PATIENT SPLIT
A performance evaluation of machine learning techniques for breast ultrasound classification	2019	UNCLEAR SPLIT
Applications of machine-learning algorithms for prediction of benign and malignant breast lesions using ultrasound radiomics signatures: A multi-center study	2022	BIRADS
Enhanced Computer-Aided Diagnosis Model on Ultrasound Images through Transfer Learning and Data Augmentation Techniques for an Accurate Breast Tumors …	2023	NOT PATIENT SPLIT
Multimodal Deep Learning Approaches to Breast Tumor Characterization using Ultrasound B-Mode and Nakagami Parametric Images	2023	2 SPLIT
Recognition of ultrasound images of breast cancer based on discrete sinc transform and deep learning	2022	NOT PATIENT SPLIT
Application of artificial intelligence ultrasound in breast cancer screening in Shigatse, Tibet	2022	NON ENGLISH
Breast lesion classification from bi modal ultrasound images by convolutional neural network	2019	DUPLICATE
Breast ultrasound image classification using a pre-trained convolutional neural network	2019	2 SPLIT
More reliable AI solution: Breast ultrasound diagnosis using multi-AI combination	2021	NOT PATIENT SPLIT
Breast Lesion Classification from Bi-Modal Ultrasound Images by Deep CNN Using Transfer and Multi-Task Learning		2 SPLIT
Deep Learning-based FemNet for Key Frame Selection in Ultrasound Videos of Breast Cancer Screening: Distilling Responsible Frames with Feature Entropy for …	2023	BIRADS
Deep-learning framework based on a large ultrasound image database to realize computer-aided diagnosis for liver and breast tumors	2021	2 SPLIT
Weakly-supervised deep learning for breast tumor segmentation in ultrasound images	2021	METRIC
Classification of breast ultrasound images in BI-RADS categories using binary decomposition strategies with convolutional neural networks	2023	2 SPLIT
Application of multiple empirical kernel mapping ensemble classifier based on self-paced learning in ultrasound-based computer-aided diagnosis for breast cancer	2021	METRIC
Breast Ultrasound Tumor Classification Using a Hybrid Multitask CNN-Transformer Network	2023	NOT PATIENT SPLIT
Deep Representation for the Classification of Ultrasound Breast Tumors.	2022	2 SPLIT
Classification of breast tumors in ultrasound using biclustering mining and neural network	2016	UNCLEAR SPLIT
Assessment of Breast Disease with a Deep Learning Model Using Bimodal Bi-View Ultrasound Images and Clinical Information: A Bi-Centre, Prospective, Diagnostic …		UNCLEAR SPLIT
Decision quality support in diagnostic breast ultrasound through Artificial Intelligence	2016	UNCLEAR SPLIT
Deep Learning-Based Breast Cancer Diagnosis at Ultrasound: Initial Application of Weakly-Supervised Algorithm Without Image Annotation Original Research	2021	2 SPLIT
c	2022	NOT PATIENT SPLIT
Explainable AI and susceptibility to adversarial attacks: A case study in classification of breast ultrasound images	2021	NOT PATIENT SPLIT
U-Net transfer learning backbones for lesions segmentation in breast ultrasound images.	2023	NOT PATIENT SPLIT
FemNet: Distilling Responsible Frames from Ultrasound Cineclips of Breast Cancer Screening Using Feature Entropy Empowered Deep Learning	2022	BIRADS

An Explainable Multi-Task Neural Network Model for Breast Cancer Detection in Ultrasound Images	2023	NOT PATIENT SPLIT
Segmentation of Breast Ultrasound Images using Densely Connected Deep Convolutional Neural Network and Attention Gates	2021	2 SPLIT
Breast Ultrasound CAD System Based on Efficient Tumour Segmentation Network and Transfer-Learned Features	2022	2 SPLIT
BUS-Net: A Fusion-based Lesion Segmentation Model for Breast Ultrasound (BUS) Images	2022	NOT PATIENT SPLIT
BUS-CAD: A computer-aided diagnosis system for breast tumor classification in ultrasound images using grid-search-optimized machine learning algorithms with extended and Boruta-selected features	2023	NOT PATIENT SPLIT
Detection and Classification of Lesions in Breast Ultrasound using a Deep Convolutional Neural Network		METRIC
Classification of Breast Ultrasound Images Based on Convolutional Neural Networks-A Comparative Study	2021	NOT PATIENT SPLIT
Fully-automated deep learning pipeline for segmentation and classification of breast ultrasound images	2022	NOT PATIENT SPLIT
Automatic semantic segmentation of breast tumors in ultrasound images based on combining fuzzy logic and deep learning—A feasibility study	2021	UNCLEAR SPLIT
Deep learning approaches for classification of breast cancer in ultrasound (US) images	2022	NOT PATIENT SPLIT
Breast lesion segmentation in ultrasound images using deep convolutional neural networks	2020	NOT PATIENT SPLIT
A study of multi-task learning with anatomical knowledge-based constraint for breast ultrasound image analysis	2023	DUPLICATE
Convolutional Neural Network Deep Learning Model for Improved Ultrasound Breast Tumor Classification	2023	NOT PATIENT SPLIT
Breast cancer segmentation and classification in ultrasound images using convolutional neural network	2021	NOT PATIENT SPLIT
End-to-end convolutional neural network framework for breast ultrasound analysis using multiple parametric images generated from radiofrequency signals	2022	2 SPLIT
A Regional-Attentive Multi-Task Learning Framework for Breast Ultrasound Image Segmentation and Classification	2023	UNCLEAR SPLIT
CF2-Net: Coarse-to-fine fusion convolutional network for breast ultrasound image segmentation	2020	2 SPLIT
Improved Classification of Ultrasound Breast Cancer RF Data with Nakagami and Derived Nakagami Parameters Using Advanced Machine Learning Algorithm	2021	DUPLICATE
Comparative Analysis of Deep Learning Networks for Lesion Classification in Breast Ultrasound Images	2023	DUPLICATE
Deep Learning Networks for Lesion Segmentation in Breast Ultrasound: A Comparative Analysis	2022	ABSTRACT ONLY
An efficient solution for breast tumor segmentation and classification in ultrasound images using deep adversarial learning	2019	NOT PATIENT SPLIT
Improving the diagnosis of breast cancer based on biophysical ultrasound features utilizing machine learning	2022	2 SPLIT
Deep meta-learning for the selection of accurate ultrasound based breast mass classifier	2022	NOT PATIENT SPLIT
Connected ResU-Net: A Deep Learning Model for Segmentation of Breast Cancer Ultrasound Images	2023	NOT PATIENT SPLIT
BreastUS: Vision Transformer for Breast Cancer Classification Using Breast Ultrasound Images	2022	NOT PATIENT SPLIT
An attention-supervised full-resolution residual network for the segmentation of breast ultrasound images	2020	2 SPLIT
Deep integrated pipeline of segmentation guided classification of breast cancer from ultrasound images	2022	NOT PATIENT SPLIT
An Automatic Segmentation of Breast Ultrasound Images Using U-Net Model	2023	NOT PATIENT SPLIT
Segmentation of Lesions from Breast Ultrasound Images using Deep Convolutional Neural Network	2021	2 SPLIT

Deep learning approach for breast ultrasound image segmentation	2021	NOT PATIENT SPLIT
Gradually Applying Weakly Supervised and Active Learning for Mass Detection in Breast Ultrasound Images	2020	UNCLEAR SPLIT
CSwin-PNet: A CNN-Swin Transformer combined pyramid network for breast lesion segmentation in ultrasound images	2023	NOT PATIENT SPLIT
ConvMixer-UNet: A Lightweight Network for Breast Lesion Segmentation in Ultrasound Images		NOT PATIENT SPLIT
Rethinking Breast Lesion Segmentation in Ultrasound: A New Video Dataset and A Baseline Network	2022	2 SPLIT
Rhaiu-net: precise tumor segmentation in breast ultrasound images	2022	UNAVAILABLE
Aquila Optimizer with Bayesian neural network for breast cancer detection on ultrasound images	2022	2 SPLIT
PSOU-net: a neural network based on improved particle swarm optimization for breast ultrasound image segmentation	2021	NOT PATIENT SPLIT
"Dr. J": An Artificial Intelligence Powered Ultrasonography Breast Cancer Preliminary Screening Solution	2020	UNCLEAR SPLIT
… 에 대한 딥러닝기반 이상영역 탐지 기술의 유효성 연구: A Study on the Effectiveness of Deep Learning based Anomaly Detection Methods for Breast Ultrasonography	2021	UNCLEAR SPLIT
Comparison of type-2 fuzzy inference method and deep neural networks for mass detection from breast ultrasonography images	2020	NOT PATIENT SPLIT
Detection of Breast Cancer in Pocket Ultrasound Images Using Deep Learning	2022	NOT PATIENT SPLIT
Convolutional Neural Networks for Breast Ultrasound Image Segmentation	2022	DUPLICATE
A method for segmentation of tumors in breast ultrasound images using the variant enhanced deep learning	2021	NOT PATIENT SPLIT
Classification for breast ultrasound using convolutional neural network with multiple time-domain feature maps	2021	NOT PATIENT SPLIT
Deep learning approaches for data augmentation and classification of breast masses using ultrasound images	2019	NOT PATIENT SPLIT
Shape-adaptive convolutional operator for breast ultrasound image segmentation	2021	UNCLEAR SPLIT
Breast tumor segmentation in ultrasound images using contextual-information-aware deep adversarial learning framework	2020	UNCLEAR SPLIT
Deep Learning-based Multi-stage segmentation method using ultrasound images for breast cancer diagnosis	2022	NOT PATIENT SPLIT
Explainable ensemble machine learning for breast cancer diagnosis based on ultrasound image texture features	2022	2 SPLIT
Bgra-net: Boundary-guided and region-aware convolutional neural network for the segmentation of breast ultrasound images	2021	2 SPLIT
A Formal validation of an Entropy-based Artificial Intelligence for Ultrasound Data in Breast Tumors	2023	TASK
Unsupervised learning method via triple reconstruction for the classification of ultrasound breast lesions	2022	2 SPLIT
… Edge-Based Selection Method for Improving Regions-of-Interest Localizations Obtained Using Multiple Deep Learning Object-Detection Models in Breast Ultrasound …	2022	DUPLICATE
Multi-task learning with context-oriented self-attention for breast ultrasound image classification and segmentation	2022	NOT PATIENT SPLIT
Ultrasound Diagnosis of Breast Masses Based on a Continuous Deep Learning Optimized Ensemble Approach: A Simulated Prospective Study and Multi-Institutional …	2022	TASK
Multi-task Learning with Consistent Prediction for Efficient Breast Ultrasound Tumor Detection	2022	2 SPLIT
Deep learning-based automatic detection of breast lesions on ultrasound images	2022	UNCLEAR SPLIT
Identifying malignant breast ultrasound images using ViT-patch	2023	NOT PATIENT SPLIT
A dual-stage transformer and MLP-based network for breast ultrasound image segmentation	2023	2 SPLIT

MFMSNet: A Multi-frequency and Multi-scale Interactive CNN-Transformer Hybrid Network for Breast Ultrasound Image Segmentation		UNCLEAR SPLIT
PERFORMANCE OF THE U-NET AND SEGNET IN THE SEGMENTATION OF BREAST ULTRASOUND LESIONS	2020	NOT PATIENT SPLIT
MCRNet: Multi-level context refinement network for semantic segmentation in breast ultrasound imaging	2022	UNAVAILABLE
A Deep Neural Network For Describing Breast Ultrasound Images in Natural Language	2022	POSTER
RCA-IUnet: a residual cross-spatial attention-guided inception U-Net model for tumor segmentation in breast ultrasound imaging	2022	NOT PATIENT SPLIT
Deep doubly supervised transfer network for diagnosis of breast cancer with imbalanced ultrasound imaging modalities	2020	UNCLEAR SPLIT
A Multiscale Nonlocal Feature Extraction Network for Breast Lesion Segmentation in Ultrasound Images	2023	NOT PATIENT SPLIT
MDA-Net: Multiscale dual attention-based network for breast lesion segmentation using ultrasound images	2022	2 SPLIT
Automation of ultrasound breast cancer images classification using deep neural networks	2022	NOT PATIENT SPLIT
Semantic Segmentation Using MSRF-NET for Ultrasound Breast Cancer	2022	UNAVAILABLE
Medical-Network (Med-Net): A Neural Network for Breast Cancer Segmentation in Ultrasound Image	2023	2 SPLIT
Breast Cancer Segmentation from Ultrasound Images Using ResNext-based U-Net Model		NOT PATIENT SPLIT
Multimodal U-Net Breast Cancer Tumor Algorithm Based on Radio Frequency (RF) Data and Ultrasound Images	2023	UNCLEAR SPLIT
A second-order subregion pooling network for breast lesion segmentation in ultrasound	2020	NOT PATIENT SPLIT
Comparative Analysis of Segment Anything Model and U-Net for Breast Tumor Detection in Ultrasound and Mammography Images	2023	NOT PATIENT SPLIT
A Multi-Resolution and Multi-Attention Location Guidance Network for Tumor Segmentation of Breast Ultrasound Image		RETRACTED
BCUIS-Net: A breast cancer ultrasound image segmentation network via boundary-aware and shape feature fusion		NOT PATIENT SPLIT
An approach based on biclustering and neural network for classification of lesions in breast ultrasound	2016	2 SPLIT
ECU-Net: multi-scale salient boundary detection and contrast feature enhancement U-Net for breast ultrasound image segmentation	2023	2 SPLIT
Breast Ultrasound Images Clustering Analysis Using Deep Clustering Method	2021	UNAVAILABLE
Adaptive lesion scale ultrasound breast image segmentation based on multi-scale attention wavelet network	2023	UNAVAILABLE
Classification Task Assisted Segmentation Network for Breast Tumor Segmentation in Ultrasound Images	2023	NOT PATIENT SPLIT
An auxiliary attention-based network for joint classification and localization of breast tumor on ultrasound images	2023	UNAVAILABLE
