# Supplementary material for: Artificial intelligence-enhanced handheld breast ultrasound for screening: A systematic review of diagnostic test accuracy
Source: PLOS Digit Health. 2025 Sep 22;4(9):e0001019. doi: 10.1371/journal.pdig.0001019 (PMC12453205; doi:10.1371/journal.pdig.0001019)
Supplement: S7 File — Data extraction results for included studies. (PDF) [file pdig.0001019.s007.pdf]

Name	Title	Task	Population	BUS Machine	Index Test	Reference Standard	Performance
Berg 2021	Impact of original and artificially improved artificial intelligence–based computer-aided diagnosis on breast US interpretation	C	**External Testing:** 638 images (orthogonal views) of 319 lesions (27.5% malignancy) from ? women (91.8% dense breasts) from a single health center in the US	Philips iU22 (98.2%); Hologic SuperSonic (1.8%)	Histological results from biopsy with benign follow-up of at least 2 years	Koios DS (Koios Medical, Inc., Chicago IL) from pre-selected ROI	0.77 AUROC of AI alone on external test set
Bunnell 2023	EARLY BREAST CANCER DIAGNOSIS VIA BREAST ULTRASOUND AND DEEP LEARNING	D	37,921 images from 2,148 women from ? clinical sites in the US (24.2% malignancy)		**Classification:** Histological results from biopsy with no record of cancer for benign **Location:** Delineations from a single radiologist	Finetuned Mask-RCNN with ResNet-101 backbone and custom heads for BI-RADS mass feature prediction.	39.2 mAP on internal test set
Byra 2019	Breast mass classification in sonography with transfer learning using a deep convolutional neural network and color conversion.	C	882 images from 882 patients (23.1% malignancy) from a single health center in California. White (69%); AANHPI (12%); Hispanic (7%); Black (5%); Native American (<1%); Other (3%) External Testing: UDIAT & OASBUD	Siemens Acuson (59%); GE L9 (21%); ATL-HDI (20%)	Histological results from biopsy with benign follow-up of at least 2 years	SVM working from finetuned VGG19 backbone pretrained on ImageNet from pre-selected ROI	0.893 AUROC on external test set
Byra 2020	Breast mass segmentation in ultrasound with selective kernel U-Net convolutional neural network.	S	882 images of 882 lesions (?% malignancy) from ? patients from a single clinical site. **External Testing:** BUSI UDIAT and OASBUD	Siemens Acuson (59%), GE L9 (21%), and ATL HDI (20%).	Delineations from a single "medical expert"	Adapted UNet with additionally dilated convolution blocks replacing traditional convolution blocks	0.701 mean DSC when *not* finetuned on external test sets
Chen 2023b	AAU-Net: An Adaptive Attention U-Net for Breast Lesions Segmentation in Ultrasound Images.	S	BUSI and UDIAT (42% malignancy) **External Testing:** STU-Hospital (?% malignancy)	LOGIQ E9 Agile; LOGIQ E9; Siemens ACUSON C512; GE Voluson E10 (100% external testing)		Adapted Unet with attention modules with varying sizes replacing traditional convolution blocks	0.802 DSC on external test set
Choi 2019	Effect of a Deep Learning Framework-Based Computer-Aided Diagnosis System on the Diagnostic Performance of Radiologists in Differentiating between Malignant and Benign	C	**External Testing:** 759 images (transverse, longitudinal, and radial) of 253 lesions from 226 patients (31.6% malignancy) from a single medical center in South Korea	Samsung Medison RS80A (100%)	**Classification:** Histological results from biopsy with benign follow-up of ?	S-Detect for Breast (Samsung Medison Co., Seongnam, Korea)	85.0% sensitivity and 95.4% specificity for CAD alone

	Masses on Breast Ultrasonography.						
Fujioka 2020	Efficient Anomaly Detection with Generative Adversarial Network for Breast Ultrasound Imaging.	C	702 images from 217 patients (48.9% malignancy in testing) from a single health center in Japan	EUB-7500; Aplio XG; Aplio 500	Histological results from biopsy with benign follow-up of at least 1 year	Bidirectional GAN from hand-cropped images	0.863 AUROC on internal test set
Fujioka 2023	Examining the effectiveness of a deep learning-based computer-aided breast cancer detection system for breast ultrasound.	D	88 videos (?% malignancy) from 45 women a single breast surgery department in Japan. **Internal Testing:** 232 videos (40.5% malignancy) from 232 women from a single breast surgery department in Japan.	Voluson E6; ACUSON S200	Location was evaluated independently by 2 experts (>10 years of experience in reading BUS). A third expert then performed adjudication.	Finetuned YOLOv3-tiny combined with edge detection post-processing of regions to isolate lesions.	95.5% sensitivity and 2.2% specificity for CAD alone
Gu 2022	Deep learning based on ultrasound images assists breast lesion diagnosis in China: a multicenter diagnostic study.	C	11,478 images from 4,149 patients (42.7% malignancy) from 30 tertiary-care hospitals in China **External Testing:** 1,291 images from 397 patients (62.1% malignancy) from 2 tertiary-care hospitals in China & BUSI	Resona7/7s/7T; Resona8/8T; DC-80	Histological results from biopsy or surgery	Finetuned VGG19 backbone pretrained on ImageNet from pre-selected ROI	0.913 AUROC on external test set
Guldogan 2023	Artificial Intelligence in BI-RADS Categorization of Breast Lesions on Ultrasound: Can We Omit Excessive Follow-ups and Biopsies?	C	**External Testing:** 1,430 orthogonal images of 715 lesions (18.8% malignancy) from 530 women	GE LOGIQ S8; GE LOGIQ E10S; ACUSON	Histological results from biopsy with benign follow-up of at least 2 years	Koios DS (Koios Medical, Inc., Chicago IL) from pre-selected ROI	98.5% sensitivity and 65.4% specificity for CAD alone
Han 2017	A deep learning framework for supporting the classification of breast lesions in ultrasound images.	C	7,408 images from 5,151 patients (42.6% malignancy) from a single health center in South Korea	Philips iU22 (70.9%); Samsung RS80A (29.1%)	Histological results from biopsy	Finetuned GoogLeNet pretrained on grayscale ImageNet from semi-automatic segmentation	0.958 AUROC on internal test set
Han 2020	Semi-supervised segmentation of lesion from breast ultrasound images with attentional generative adversarial network.	S	2,800 images from 2,800 patients (50% malignancy) from a single hospital in China External Testing: UDIAT	Philips iU22 (100% training); Siemens ACUSON Sequoia C512 (100% testing);	Delineations from physicians at department of US	GAN-based architecture with attention and segmentation mask generator and discriminator networks	0.78 DSC on external test set
Hassanien 2022	Transformer-Based Radiomics for Predicting Breast Tumor Malignancy Score in Ultrasonography	C	UDIAT	Siemens ACUSON Sequoia C512		Finetuned SwinTransformer from hand-cropped images	0.93 AUROC on internal test set

Huang 2022a	Boundary-rendering network for breast lesion segmentation in ultrasound images.	S	2,020 images from ? patients (50.2% malignancy) from UDIAT and a single hospital in China	Mindray Resona 7 (77.1%); Siemens ACUSON Sequoia (8.1%); HDI 5000 SonoCT (14.8%)	Delineations from “experienced radiologist”	Combination CNN and graph convolutional architecture for mask and specific boundary-rendering, respectively.	0.919 DSC on patient-wise 5-fold CV
Huang 2022b	Extracting keyframes of breast ultrasound video using deep reinforcement learning.	F	2,606 videos from 653 patients (26.7% malignancy) from 8 hospitals in China	Philips; Mindray; Toshiba	**Keyframe:** Frame from “experienced sonographers” **Location:** Bounding box and BI-RADS indicators from “experienced sonographers” **Classification:** Histological results from biopsy or surgery	Reinforcement learning scheme with 3D convolutional BiLSTM with frame-based reward structure based on lesion presence, proximity to labelled frame, and malignancy indicators.	0.846 diagnostic AUROC on internal test set from selected frames
Karlsson 2022	Machine learning algorithm for classification of breast ultrasound images	C	BUSI **External Testing**: 293 images from ? women (90.1% malignancy) from a single university hospital in Sweden	LOGIQ E9 (training); LOGIQ E9 Agile (training)		Finetuned ResNet50V2 from hand- and automaticallly-cropped images	0.81 AUROC on external test set
Kim 2021b	Weakly-supervised deep learning for ultrasound diagnosis of breast cancer.	D	1,400 images from 971 patients (50% malignancy) from a single university hospital in South Korea External Testing: 200 images from 125 patients (50% malignancy) from a single university hospital in South Korea	Philips (100% training, 8% testing); GE (84% testing); Siemens (8% testing)	Histological results from biopsy with benign follow-up of at least 2 years. Location from single radiologist	GoogLeNet from hand-cropped images	0.9 AUROC on external test set
Lai 2022	Evaluation of physician performance using a concurrent-read artificial intelligence system to support breast ultrasound interpretation.	D	**External testing:** 344 images from 172 women (37.8% malignancy) from a single hospital in Taiwan	Philips; Toshiba/Canon	Histological results from biopsy with benign follow-up of at least 2 years	BU-CAD (TaiHao Medical Inc., Taipei Taiwan)	0.838 AULROC on external test set
Lee 2022	Differing benefits of artificial intelligence-based computer-aided diagnosis for breast US according to workflow and experience level.	C	**External testing:** 492 lesions from 472 women (40.7% malignancy) from a single health center in South Korea	Samsung RS80A (100%)	Histological results from biopsy with benign follow-up of at least 2 years	S-Detect for Breast (Samsung Medison Co., Seongnam, Korea)	0.855 AUROC on external test set

Liao 2023	Artificial intelligence-assisted ultrasound image analysis to discriminate early breast cancer in Chinese population: a retrospective, multicentre, cohort study.	C	15,910 images from 6,795 patients (2.56% malignancy) from a single hospital in China **External Testing 1:** 896 images from 391 patients (2.23% malignancy) from a single hospital in China **External Testing 2:** 490 images from 235 patients (2.04% malignancy) from a single hospital in China	Philips EPIQ7 (50% training); GE E9 (30% training, 72.7% testing); Siemens (10% training); Mindray R9 (10% training); Philips EPIQ5 (27.3% testing)	Histological results from biopsy with benign follow-up of at least 3 years	80 Dual-branch ResNet50 learners for B-mode and Doppler ensembled into parent model from ROI	0.956 AUROC on external test set
Meng 2023	DGANet: A Dual Global Attention Neural Network for Breast Lesion Detection in Ultrasound Images.	D	7,040 images from 3,759 women (60.7% malignancy) from ? hospitals in China **External testing:** BUSI	Siemens; Philips; Aloka; Hitachi	Histological results from biopsy	Adapted YOLOv3 with added bilateral spatial and global channel attention modules	0.840 mAP on internal test set
Ning 2022	SMU-Net: Saliency-Guided Morphology-Aware U-Net for Breast Lesion Segmentation in Ultrasound Image.	S	UDIAT, BUSI, and ultrasoundcases**External testing:** onlinemedicalimages and OASBUD	ACUSON Sequoia C512; LOGIQE 9; LOGIQ E9 Agile; Hitachi; Philips iU22 (47.5% external testing); SonixTouch L14 (52.5% external testing)		Custom Unet with background/foreground information streams and shape-, edge-, and position-aware fusion units.	0.872 DSC on external test set
Park 2019	A computer-aided diagnosis system using artificial intelligence for the diagnosis and characterization of breast masses on ultrasound	C	**External testing:** 100 video clips of lesions from 91 women (41% malignant) from a single hospital in South Korea	Samsung RS80A (100%)	Histological results from biopsy or surgery	S-Detect for Breast (Samsung Medison Co., Seongnam, Korea)	`+0.105 difference in AUROC with/without CAD for included readers on external test set
Qiu 2023	Prospective assessment of breast lesions AI classification model based on ultrasound dynamic videos and ACR BI-RADS characteristics.	D	480 video clips (18,122 images) of 480 lesions from 420 women (40.8% malignancy) from a single hospital in China **Prospective testing:** 292 video clips of 292 lesions from 278 women (42.5% malignancy) from two hospitals in China	Canon Toshiba Apogee 6800; Siemens S3000	**Location:** two "experienced radiologists" independently segmented lesions **Classification:** histological results from biopsy	Finetuned YOLOv5 network with attention	0.87 AUROC on prospective testing set

Qu 2020	An attention-supervised full-resolution residual network for the segmentation of breast ultrasound images.	S	980 images from 980 women (60.7% malignancy) from a single university hospital in China and UDIAT	Philips iU22 xMATRiX; LOGIQ E9; ACUSON Sequoia	Labeled by "experts"	Custom ResNet with varying-scale attention modules and upsampling	0.905 DSC on five-fold cross-validation
Shen 2021	Artificial intelligence system reduces false-positive findings in the interpretation of breast ultrasound exams.	C	5,442,907 images from 143,203 patients (1.1% malignancy) from >100 hospitals in New York **External Testing:** BUSI	Affiniti 70G (37.1%); S1000 (19.4%); S3000 (13.9%); S2000 (11.9%); Other (17.7%)	Histological results from biopsy with benign follow-up of at most 15 months (test set) Pathology report (training set)	Deep convolutional network with spatial and scan-wise attention and saliency map concatenation from entire input image set per breast	0.976 AUROC on internal test set
Wanderley 2023	Application of artificial intelligence in predicting malignancy risk in breast masses on ultrasound	C	**External testing:** 555 lesions from 509 women (40% malignancy) from a single health center in Brazil		Histological results from biopsy	Koios DS (Koios Medical, Inc., Chicago IL) from pre-selected ROI	98.2% sensitivity and 39.0% specificity of CAD alone on external test set
Wang 2021	Residual feedback network for breast lesion segmentation in ultrasound image	S	3,279 images from 1,154 patients (57.2% malignancy) (ultrasoundcases & BUSI) **External Testing:** BUSI & radiopaedia			Custom UNet with ResNet34 encoder and residual feedback	0.82 DSC on external test set
Webb 2021	Comparing deep learning-based automatic segmentation of breast masses to expert interobserver variability in ultrasound imaging.	S	31,070 images from 851 women (?% malignancy) from a single clinic in the USA		Segmented by 3 "experts" (testing) and "research technologists" with experience in US (development)	Custom DenseNet264 with added feature pyramid network and ResNet-C input stream pretrained on thyroid US images	0.832 DSC on internal test set
Wu 2022	A Comparative Study of Multiple Deep Learning Models Based on Multi-Input Resolution for Breast Ultrasound Images.	C	13,684 images from 3,447 patients (28.7% malignancy) from a single hospital in China External Testing: 440 images from 228 patients (54.3% malignancy) from a single hospital in China	GE Logiq E9 (33%); Philips EPIQ 5 (33%); Mindray Resonan 7 (33%)	Histological results from biopsy or surgery	Finetuned MobileNet from hand-cropped images	0.893 AUROC on external test set

Xiang 2023	Deep Learning-assisted Diagnosis of Breast Lesions on US Images: A Multivendor, Multicenter Study.	C	39,899 images of 8,051 lesions from 7,218 patients (64.1% malignancy) from a single univesity hospital in China **External testing 1:** 2,637 images of 777 lesions from 693 patients (47.6% malignancy) from a single hospital in China **External testing 2:** 957 images of 419 lesions from 382 patients (48.9% malignancy) from a single hospital in China **External testing 3:** 2,416 images of 648 lesions from 504 patients (25.3% malignancy) from a single hospital in China		Histological results from biopsy or surgery	Custom finetuned DenseNet121 with self-attention averaged over all views of a lesion	0.91 AUROC on external test set
Zhang 2023	Fully automatic tumor segmentation of breast ultrasound images with deep learning.	S	1,342 images from ? patients from 5 hospitals in China External Testing: 570 images from ? patients from a single hospital in China & BUSI & onlinemedicalimages		Delineations from “experienced radiologists”	Fully-convolutional network with VGG16 backbone from pre-selected ROI	0.89 mean IOU on external test set
Zhao 2022	Focal U-Net: A Focal Self-attention based U-Net for Breast Lesion Segmentation in Ultrasound Images.	S	9,836 images from 4,875 patients from ? hospitals in China	SIEMENS; PHILIPS; ALOKA; HITACHI	Delineations are from 3 “experienced radiologists”	Custom U-Net architecture with local and de-noising attention	0.838 DSC on internal test set
Zhuang 2019	An RDAU-NET model for lesion segmentation in breast ultrasound images.	S	857 images from ? patients from a single hospital in the Netherlands (ultrasoundcases) External Testing: STU-Hospital & UDIAT			Custom attention-based residual Unet	0.834 DSC on external test set
