# Supplementary material for: Artificial intelligence-enhanced handheld breast ultrasound for screening: A systematic review of diagnostic test accuracy
Source: PLOS Digit Health. 2025 Sep 22;4(9):e0001019. doi: 10.1371/journal.pdig.0001019 (PMC12453205; doi:10.1371/journal.pdig.0001019)
Supplement: S8 File — For each included study, results from the QUality Assessment of Diagnostic Accuracy Studies-2 (QUADAS-2) criteria used for this review. (PDF) [file pdig.0001019.s008.pdf]

## Complete QUADAS-2 Bias Assessment Results (Post Discussion)

See S3 for list of questions. Y = Yes; N = No; U = Unclear; H = High; L = Low; NA = Not Applicable.

| | Q1 | Q2 | Q3 | Q4 | Q5 | Q6 | Q7 | Q8 | Q9 | Q10 | Q11 | Q12 | Q13 | Q14 | Q15 |
|---|---|---|---|---|---|---|---|---|---|---|---|---|---|---|---|
| Berg 2021 | N | Y | Y | H | N | Y | NA | N | H | Y | Y | L | N | N | Y |
| Byra 2019 | U | N | Y | H | Y | Y | N | N | H | Y | Y | H | N | N | Y |
| Choi 2019 | Y | N | N | H | Y | Y | Y | N | H | N | Y | H | N | N | N |
| Fujioka 2020 | U | N | N | H | U | Y | NA | NA | H | N | Y | H | N | N | N |
| Gu 2022a | U | N | Y | H | Y | Y | NA | Y | H | N | Y | H | N | N | N |
| Guldogan 2023 | Y | Y | Y | H | Y | Y | Y | NA | H | N | Y | H | N | N | Y |
| Han 2017 | U | Y | N | H | Y | Y | NA | NA | H | N | NA | H | N | N | N |
| Hassanien 2022 | U | U | N | H | Y | Y | N | NA | H | N | Y | U | N | N | Y |
| Karlsson 2022 | U | U | Y | H | Y | Y | N | NA | H | N | Y | H | Y | N | Y |
| Lee 2022 | Y | Y | Y | H | Y | Y | Y | N | H | Y | Y | L | N | N | Y |
| Liao 2023 | Y | N | Y | H | Y | Y | N | N | H | Y | Y | L | Y | N | N |
| Park 2019 | U | N | Y | H | Y | Y | Y | N | H | N | Y | H | Y | Y | N |
| Shen 2021 | U | Y | Y | L | Y | Y | N | Y | H | N | Y | L | H | H | N |
| Wanderley 2023 | N | U | Y | H | Y | Y | N | N | H | N | Y | H | Y | Y | N |
| Wu 2022 | U | N | Y | H | Y | Y | N | N | H | N | Y | H | N | N | N |
| Xiang 2023 | U | Y | Y | H | Y | Y | N | N | H | N | Y | H | Y | Y | Y |
| Byra 2020 | U | U | Y | H | N | Y | NA | NA | H | N | Y | H | NA | U | Y |
| Chen 2023 | N | Y | Y | H | Y | Y | NA | NA | H | N | Y | H | NA | U | Y |
| Han 2020b | U | Y | Y | H | Y | Y | NA | NA | H | N | Y | H | Y | U | N |
| Huang 2022a | N | U | N | H | U | Y | NA | NA | H | N | Y | H | NA | U | N |
| Ning 2022 | U | U | Y | H | Y | Y | NA | NA | H | N | Y | H | NA | U | N |
| Qu 2020 | U | Y | Y | H | Y | Y | NA | NA | H | N | Y | U | NA | U | Y |
| Wang 2021 | U | U | Y | H | Y | Y | N | NA | H | N | Y | H | NA | U | N |
| Webb 2021 | U | N | N | H | N | N | NA | N | H | Y | N | H | NA | U | Y |
| Zhang 2023 | U | U | Y | H | Y | Y | N | NA | H | N | Y | H | NA | U | N |
| Zhao 2022 | U | U | N | H | Y | Y | N | NA | H | Y | Y | L | N | U | N |
| Zhuang 2019 | U | U | Y | H | Y | Y | N | NA | H | N | Y | H | N | U | N |
| Bunnell 2023 | N | N | N | H | Y | Y | NA | NA | H | N | Y | H | N | N | Y |
| Fujioka 2023 | U | U | N | H | Y | Y | N | N | L | N | Y | H | N | N | Y |
| Kim 2021 | Y | U | Y | H | Y | Y | N | NA | H | N | Y | H | N | N | Y |
| Lai 2022 | U | Y | Y | H | Y | Y | Y | N | H | U | Y | H | N | N | Y |
| Meng 2023 | U | U | Y | H | Y | Y | N | NA | H | N | Y | H | Y | Y | Y |
| Qiu 2023 | U | N | Y | H | Y | Y | NA | N | L | N | Y | U | Y | Y | N |
| Huang 2022b | U | U | N | H | Y | Y | NA | N | L | N | Y | H | NA | U | N |
