# Supplementary material for: Artificial intelligence-enhanced handheld breast ultrasound for screening: A systematic review of diagnostic test accuracy
Source: PLOS Digit Health. 2025 Sep 22;4(9):e0001019. doi: 10.1371/journal.pdig.0001019 (PMC12453205; doi:10.1371/journal.pdig.0001019)
Supplement: Attachment — Submitted filename: Response to Reviewers.docx [file pdig.0001019.s009.docx]

We thank the reviewers and the editor for their thoughtful comments on our manuscript. We have carefully considered each of the raised points and prepared point-by-point responses below.

**Response to Reviewer 1**

**Comment 1:** Consider clarifying terminology for a broader digital health audience—for example, explain “Dice Similarity Coefficient” briefly where first mentioned.

**Response 1:** In response, we have updated the paragraph defining the metrics with short definitions for AUROC, AP, DSC, and IOU:

“Lesion detection AI is evaluated by average precision (AP) or mean average precision (mAP). Both mAP and AP represent the area under the precision-recall curve, quantifying identification of true lesions balanced with prediction of false positive lesions Frame selection is evaluated by AUROC in frame selection and/or diagnosis from selected frames. Cancer classification is evaluated by AUROC or sensitivity/specificity. AUROC is a rank-based metric which conveys the probability that a randomly selected malignant lesion will have a higher predicted probability of cancer than any random benign lesion. Lesion segmentation is evaluated by Dice Similarity Coefficient (DSC) or intersection over union (IOU). DSC is equal to 2× the total overlapping area of two lesion segmentations, divided by the total combined area of the lesion segmentations. IOU is defined as the total overlapping area of two lesion segmentations divided by the area of their union.”

**Comment 2:** While limitations are acknowledged, consider emphasising how the high prevalence of malignancy in most studies (unrealistic for screening populations) may distort diagnostic performance.

**Response 2:** In response, we have added the following sentence in the **Main findings** section: “Models trained on datasets enriched with malignancies may require an additional calibration step for use in the general population.” We have also strengthened our statements about dataset representation to explicitly call out cancer prevalence: “However, researchers developing AI-informed BUS systems should concentrate their efforts on providing explicit, high-quality model validation on geographically external test sets, with breast cancer prevalence representative of screening, with complete metadata.”

**Comment 3:** Another potential limitation of this review is the restricted number of databases used in the literature search (PubMed and Google Scholar only). While these are important sources, the exclusion of other major databases such as Embase, Scopus, or Web of Science might have led to the omission of relevant studies, particularly from non-biomedical domains or regional sources. For future systematic reviews in this fast-evolving AI landscape, expanding the database scope is recommended to ensure a more exhaustive literature capture.

**Response 3:** Thank you for this feedback. We believe the most relevant literature is well-covered by the databases we used, but in the future it would be good to include these other databases.

**Comment 4:** Enhance the self-sufficiency of figure legends so that readers can interpret the figure without needing to cross-reference the text.

**Response 4:** In response to this comment, we have updated all figure captions to be more self-contained. See the updated captions below.

Fig. 1. PRISMA selection diagram. PRISMA 2020 flow diagram showing study selection and screening process from PubMed and Google Scholar for perception (frame selection, lesion detection, and segmentation) and interpretation (cancer classification) breast ultrasound (BUS) AI. HHBUS = handheld breast ultrasound; NAC = neoadjuvant chemotherapy; LNM = lymph node metastasis.

Fig. 2. Performance of lesion classification studies. Scatter plot showing reported performance (as measured by AUROC) for lesion classification (interpretation) studies against the reported size of the development dataset by number of breast ultrasound images. Studies are additionally identified by whether reported performance is on an internal or external testing set. Internal testing sets are sampled from the same underlying population as the development set.

Fig. 3. Process diagram showing clinical application time and AI task types of studies. Diagram showing the different opportunities in the care paradigm where AI can be applied in a low-resource or teleradiology-first setting. Agents include the patient and healthcare team (sonographer and/or radiologist). Clinical decisions are lesion diagnosis or localization. Studies are classified as being interpretation (cancer classification) or perception (frame selection, lesion detection, and segmentation) according to their AI task type (one of: real-time detection, offline detection, frame selection, classification and/or segmentation).

Fig. 4. QUADAS-2 bias assessment results. QUality Assessment of Diagnostic Accuracy Studies-2 (QUADAS-2) bias assessment results. Figure is best viewed in color. Studies are assessed for risk of bias in patient selection, index test (i.e., AI model) definition, reference standard (ground truth) definition, and the flow and timing of their study in relation to decisions about patient care. Studies are assessed for concerns about applicability for patient selection, index test definition, and reference standard definition. Reference standard assessments for frame selection studies are reported classification first, frame selection second. H = high; U = unclear. The full QUADAS-2 criteria adapted from (11) is available in the S3.

Fig. 5. Diversity reporting completeness heatmap. Heatmap showing axes of reported diversity for included studies. Studies are evaluated based on completeness of reporting on the racial/ethnic, age, breast density, background echotexture, and body mass index diversity of their participants, as well as included breast ultrasound machine types. Figure is best viewed in color. Studies which fail to report along any of the included axes are omitted from the plot. Studies which only use one kind of ultrasound machine and report on an additional axis are indicated with a – on the above plots.

**Comment 5:** Consider recommending more explicitly the development of a shared benchmark dataset for AI-informed BUS, which could accelerate field-wide progress.

**Response 5:** This is a good idea. We have added the following sentence into the final paragraph of the manuscript: “Creation of a secure benchmark dataset which meets these criteria is one promising method by which new models can be evaluated, and this would be helpful in advancing the field.”

**Response to Reviewer 2**

**Comment 1:** The introduction would benefit from a clearer framing of handheld ultrasound's role in breast cancer screening and why AI is needed. For instance, the authors can consider citing evidence that even without AI, handheld ultrasound can achieve high cancer detection sensitivity in low-resource settings. This underscores ultrasound's potential as an alternative to mammography in these regions.

**Response 1:** In response, we have added the following sentence and references to the Introduction section. “Preliminary evidence from BUS screening programs in LMICs have demonstrated promising sensitivity (9, 10).”

9) Akbari ME, Haghighatkhah H, Shafiee M, Akbari A, Bahmanpoor M, Khayamzadeh M. Mammography and ultrasonography reports compared with tissue diagnosis-an evidence based study in Iran, 2010. Asian Pacific Journal of Cancer Prevention. 2012;13(5):1907-10.

10) Dan Q, Zheng T, Liu L, Sun D, Chen Y. Ultrasound for breast cancer screening in resource-limited settings: current practice and future directions. Cancers. 2023;15(7):2112.

**Comment 2:** The search strategy (PubMed and Google Scholar up to Dec 2023) is appropriate, but the authors should clarify if any other databases (e.g., EMBASE or IEEE Xplore) or gray literature were considered to capture relevant studies from both clinical and technical fields. Some AI studies appear only at conferences.

**Response 2:** Thank you for this feedback. Google Scholar indexes all the most prominent machine learning and AI conferences (NeurIPS, ICLR, ICML, MICCAI, and more) as well as gray literature repositories including arXiv and OpenReview, so we are confident that we have captured the major publications in this area. However, we will include these other databases in the future.

**Comment 3:** In the Methods, the manuscript groups studies by “AI task type, application time, and AI task.” For readers less familiar with AI jargon, the authors can define what is meant by frame selection, detection, segmentation, and classification tasks in the context of breast ultrasound.

**Response 3:** In response, we have added the following explanations of the AI tasks into the Data synthesis subsection of the Methods section: “In brief for this review, lesion segmentation is the pixel-wise delineation of the border of a breast lesion in a BUS exam frame which is known to have a lesion. Lesion detection is the localization of a lesion (surrounding the lesion with a bounding box) in a BUS exam frame which is not known to contain a lesion a priori. Frame selection is the filtering of BUS exam frames to those which are most informative or most likely to contain a lesion. Cancer classification is the prediction of whether a given BUS exam frame or lesion is malignant.”

**Comment 4:** The result that 79% of the studies were at high or unclear risk of bias is a crucial finding. The manuscript should elaborate on the main sources of bias observed. Were most studies lacking a proper independent validation cohort (index test bias), or were there concerns about patient selection (e.g., retrospective studies using case–control designs), or reference standard issues?

**Response 4:** We have added the following into the Discussion’s Main findings section: “We find that 79% of included studies are at high or unclear risk of bias. The main sources of bias observed were: (1) unclear source of ground truth for lesion location; (2) incomplete reporting of the patient/image selection process; and (3) failure to aggregate image-level results into exam- or woman-level predictions.” See also the response to Comment #5 below.

**Comment 5:** Following on the previous note, although the Discussion touches on the lack of robust evidence, it could more explicitly highlight the limitations of the studies reviewed. Key issues to discuss include: (a) the dearth of external validation (b) small or homogeneous datasets (c) spectrum and setting (many studies used datasets enriched with cancer cases or conducted retrospective analysis of clinical exams, which may overestimate performance compared to a true screening scenario with low prevalence); and (d) inconsistent reporting different studies use different performance metrics and thresholds (some report AUC, others report sensitivity at fixed specificity), which complicates direct comparison.

**Response 5:** In response to this comment, as well as comments 4, 6, and 7, we have added a new paragraph into the end of the Discussion’s Main findings section. The paragraph is copied below.

“We find that 79% of included studies are at high or unclear risk of bias. The main sources of bias observed were: (1) unclear source of ground truth for lesion location; (2) incomplete reporting of the patient/image selection process; and (3) failure to aggregate image-level results into exam- or woman-level predictions. Furthermore, the lack of external model validation, on imaging from new populations of women at different institutions, is a key weakness of the current literature in AI-enhanced BUS. Prospective validation on a racial/ethnically diverse, external population of women represents the gold standard in model evaluation. None of the included studies perform this style of validation. Lack of reporting on data diversity is also a concern, limiting evidence for model generalizability. While the majority of studies report patient age and BUS machine types, very few studies report on patient BMI, racial/ethnic distribution, breast density, and background echotexture (Byra 2019, Gu 2022, Shen 2021, and Kim 2021).”

**Comment 6:** Nearly all the included studies seem to have evaluated AI algorithms on internal datasets or via cross-validation. This is a well-recognized shortcoming in AI diagnostics literature, as models often perform worse on truly independent data due to overfitting or population differences. The manuscript should stress that without external multi-center validation (ideally using prospective cohorts), the reported high performance of AI cannot be assumed to hold in new populations.

**Response 6:** In response, we have added the following into the Discussion’s Main findings section: “Furthermore, the lack of external model validation, on imaging from new populations of women at different institutions, is a key weakness of the current literature in AI-enhanced BUS. Prospective validation on a racial/ethnically diverse, external population of women represents the gold standard in model evaluation. None of the included studies perform this style of validation.” See also the response to Comment #5 above.

**Comment 7:** In line with generalizability, the authors should consider commenting on the diversity (or lack thereof) in the data used by the AI models. Did the review find that most models were trained on data from a single country or a single manufacturer’s equipment?

**Response 7:** Figure 5 and the corresponding discussion of the heatmap in the Study quality assessment section of the manuscript address the reviewer’s comment. In response, we have also added the following into the Discussion’s Main findings section: “Lack of reporting on data diversity is also a concern, limiting evidence for model generalizability. While the majority of studies report patient age and BUS machine types, very few studies report patient BMI, racial/ethnic distribution, breast density, and background echotexture (Byra 2019, Gu 2022, Shen 2021, and Kim 2021).” See also the response to Comment #5 above.

**Comment 8:** A critical aspect to address is how applicable the reviewed studies are to real-world screening scenarios (as opposed to diagnostic settings). The inclusion criteria captured studies on AI for BUS, but it’s not always clear if these studies simulated a screening context (meaning, asymptomatic women in population screening) or if they were focused on classifying known lesions in a clinical setting.

**Response 8:** Yes, this is an important source of bias in some of the datasets which would limit the applicability of the models to screening. The following sentence has been added: “Risk of bias in participant selection was also high due to unrepresentative dataset composition; only two studies (Liao 2023 and Shen 2021) trained or validated their AI methods on datasets with screening cancer prevalence (<3%).”

**Comment 9:** The Discussion should include a perspective on the clinical readiness of AI-informed handheld BUS tools. Despite many studies reporting high diagnostic accuracy, none of the AI systems reviewed appear to have been widely deployed in routine screening practice yet. It would be useful if the authors commented on what barriers remain before these AI tools can be used in the field. For instance, do any of the reviewed models have regulatory approval (like FDA clearance)? Are there ongoing prospective trials or implementation studies for AI-assisted ultrasound screening?

**Response 9:** In response, we have added the following text into the Main findings section of the discussion: “Included studies examined the following commercial systems, as well as 25 academic models: S-Detect for Breast (Samsung Medison Co., Seongnam, Korea) (4 studies), CADAI-B (BeamWorks Inc., Daegu, Korea) (1 study), BU-CAD (TaiHao Medical Inc., Taipei City, Taiwan) (1 study), and Koios DS (Koios Medical, Inc., Chicago IL) (3 studies). Koios DS is the only system included in this review with current US FDA clearance.”

**Comment 10:** The authors have cited many pertinent studies; however, to strengthen the scholarly foundation, a few additional recent works could be referenced. For instance, Brot (Ultrasonography, 2024) provides a comprehensive review of AI in breast ultrasound and similarly conclude that more prospective studies are needed, and that AI could play a significant role in low-income regions.

**Response 10:** We have added the requested citation into the Comparison with other studies section.

**Comment 11:** Terminology: Use consistent terminology when referring to AI throughout the manuscript. Phrases like “AI-informed BUS” vs “AI-enhanced BUS” appear; it may be better to stick with one term (perhaps "AI-enhanced ultrasound") after initially defining it.

**Response 11:** In response, we have harmonized terminology in the manuscript to refer to “AI-enhanced” BUS only.

**Comment 12:** The Conclusion section might be made slightly more cautious. Currently, the abstract concludes there has been “encouraging development,” but evidence “lacks robustness,” and that high-quality validation is key. As this work shows, while AI for handheld BUS shows promise and could be transformative for global breast cancer screening, substantial further research is required to confirm its accuracy and effectiveness in real-world screening programs. This tempered conclusion will ensure the stakeholders in global health do not take the current performance at face value.

**Response 12:** The conclusion section of the abstract has been updated to the following: “However, despite studies demonstrating high performance, substantial further research is required to validate reported performance in real-world screening programs. High-quality model validation on geographically external, screening datasets will be key to realizing the potential for AI-enhanced BUS in increasing screening access in resource-limited environments.”

**Response to Reviewer 3**

**Comment 1:** This systematic review evaluates AI-enhanced handheld breast ultrasound (BUS) for cancer screening, particularly in low-resource settings. They do an excellent job classifying the tasks involved (framing, segmentation, interpretation), and find that while AI models show promising performance in tasks like lesion detection and classification, many studies lack robust validation on external datasets, limiting generalizability. The review emphasizes the need for high-quality validation, especially on diverse populations. Despite encouraging results, the authors conclude that further development and external validation are necessary before AI-enhanced BUS can be widely implemented in screening programs. Spot on! And the authors do it succinctly.

**Response 1:** We thank the reviewer for their enthusiasm.

**Response to Journal Requirements:**

**Comment 1:** Please provide a complete Data Availability Statement in the submission form, ensuring you include all necessary access information or a reason for why you are unable to make your data freely accessible. If your research concerns only data provided within your submission, please write "All data are in the manuscript and/or supporting information files" as your Data Availability Statement.

**Response 1:** We have provided the statement as requested.

**Comment 2:** Please provide separate figure files in .tif or .eps format.

**Response 2:** We have provided the materials as requested.

**Comment 3:** As required by our policy on Data Availability, please ensure your manuscript or supplementary information includes the following: A numbered table of all studies identified in the literature search, including those that were excluded from the analyses. For every excluded study, the table should list the reason(s) for exclusion. If any of the included studies are unpublished, include a link (URL) to the primary source or detailed information about how the content can be accessed. A table of all data extracted from the primary research sources for the systematic review and/or meta-analysis. The table must include the following information for each study: Name of data extractors and date of data extraction Confirmation that the study was eligible to be included in the review. All data extracted from each study for the reported systematic review and/or meta-analysis that would be needed to replicate your analyses. If data or supporting information were obtained from another source (e.g. correspondence with the author of the original research article), please provide the source of data and dates on which the data/information were obtained by your research group. If applicable for your analysis, a table showing the completed risk of bias and quality/certainty assessments for each study or outcome. Please ensure this is provided for each domain or parameter assessed. For example, if you used the Cochrane risk-of-bias tool for randomized trials, provide answers to each of the signalling questions for each study. If you used GRADE to assess certainty of evidence, provide judgements about each of the quality of evidence factor. This should be provided for each outcome. An explanation of how missing data were handled. This information can be included in the main text, supplementary information, or relevant data repository. Please note that providing these underlying data is a requirement for publication in this journal, and if these data are not provided your manuscript might be rejected.

**Response 3:** We have provided the materials as requested. See new	supplemental documents S6, S7, and S8.
